# Effects of breathing re-education on endurance, strength of deep neck flexors and pulmonary function in patients with chronic neck pain: A randomised controlled trial

**DOI:** 10.4102/sajp.v78i1.1611

**Published:** 2022-04-26

**Authors:** Sahreen Anwar, Syed A. Arsalan, Hamayun Zafar, Ashfaq Ahmed, Syed A. Gillani, Asif Hanif

**Affiliations:** 1 Department of Physical Therapy, Faculty of Rehabilitation Sciences, University of Lahore, Lahore, Pakistan; 2 Department of Physical Therapy, Faculty of Rehabilitation Sciences, King Saud University, Riyadh, Saudi Arabia; 3 Department of Biostatistics, Faculty of Rehabilitation Sciences, University of Lahore, Lahore, Pakistan

**Keywords:** endurance, neck pain, breathing, re-education, pulmonary function

## Abstract

**Background:**

People with chronic neck pain show decreased endurance and strength of cervical muscles with compromised respiratory function. There is little evidence that improvement in breathing function of people with neck pain can help in enhancing cervical muscle strength and pulmonary function. The objective of this our clinical trial was to examine the effects of breathing re-education combined with physiotherapy on endurance and strength of deep neck flexors, and pulmonary function in patients with chronic neck pain.

**Methods/design:**

In this double blind randomised clinical trial, 30 patients with chronic neck pain (25–50 years old) were randomly allocated to two groups. Group A, physiotherapy (*n* = 15), and Group B, breathing re-education (*n* = 15). The duration of intervention was eight weeks with treatment five days a week. The endurance was measured with the craniocervical flexion test, strength with a handheld dynamometer (Baseline USA) and pulmonary functions with the Spiro lab 4 (USA) at baseline, at week four and at week eight of the intervention.

**Discussion:**

There was a significant between group improvement in the strength of deep neck flexors and forced vital capacity (FVC) in Group B *p* = 0.0001 and *p* = 0.0200 (*p* ˂ 0.05) respectively. Intergroup comparisons showed no significant differences for endurance, cervical extensor strength, Forced Expiratory Volume in one second (FEV1), and FEV1/FVC percentage.

**Conclusion:**

Our study concluded that breathing re-education combined with other physiotherapy management is effective for improving the strength of neck flexors and increasing FVC in people with chronic neck pain.

**Clinical implication:**

Breathing re-education may be part of physiotherapy management in patients with chronic neck pain.

**Trial Registration:**

Iranian Registry of Clinical Trials, IRCT20200226046623N1, https://www.irct.ir/trial/46240.

## Introduction

Chronic neck pain is a common disability and is an important health problem in the general population. A systematic analysis of the global burden of disease reveals that the annual prevalence of neck pain is increasing with the highest burden in Europe, Africa and the Middle East (Safiri et al. 2020). There is inhibition of the deep cervical flexors (longus colli, longus capitis), whereas the superficial neck flexors (sternocleidomastoid, anterior scalene) are overactivated in people with chronic neck pain (Falla, Jull & Hodges 2004). During different head and neck movements, the clavicle and the manubrium sterni are elevated expanding the rib cage, an important feature of inspiration. In people with chronic neck pain, this expansion of the thoracic cage becomes compromised due to muscular imbalance, postural changes and segmental instability leading to shallow breathing which ultimately leads to respiratory dysfunction (Dimitriadis et al. 2013; Kaur, Pattnaik & Mohanty 2013).

According to a review there is a decline in the partial pressure of the arterial carbon dioxide and maximal voluntary ventilation in people with chronic neck pain and the strength of respiratory muscles and chest mechanics is also compromised (Dimitriadis et al. 2016). Many factors have a negative influence on normal respiratory function like decreased endurance and strength of the deep neck flexors, hyperactivity and increased fatigability of the superficial neck flexors, limitation of the range of motion of the neck, decrease in the proprioception and disturbances in the neuromuscular control of the neck (Cheon et al., 2020; Kirthika et al. 2018). Thus, it is reasonable to speculate that in the presence of chronic neck pain, the respiratory function of patients is compromised, so a multimodal treatment approach is needed to treat chronic neck pain and its associated disorders (Blanpied et al. 2017).

Breathing techniques have been part of chronic pain syndrome treatment programmes as one of a range of relaxation techniques (Busch et al. 2012). Individuals with chronic neck pain have altered breathing patterns and breathing re-education has an immediate positive effect on cervical muscle activation and respiratory function (Kapreli, Vourazanis & Strimpakos 2008; Yeampattanaporn et al. 2014). Breathing exercises aid in inhibiting the use of accessory muscles and stimulate primary muscles of respiration, thus preventing compensation in superficial and deep cervical muscles. Kang, Jeong and Choi (2016) investigated the role of sternocleidomastoid and scaleni as accessory muscles of respiration by implementing breathing exercises in people with chronic neck pain. There were significant differences in the activities of the sternocleidomastoid and the scaleni, and the Neck Disability Index in the control and the experimental groups. The between-group analysis also shows significant differences in the activity of the sternocleidomastoid and Neck Disability Index after implementation of breathing exercises (Kang et al. 2016).

Recently studies have been conducted with people with chronic neck pain and its relevance to respiratory dysfunction, but it is difficult to draw any conclusion from them, due to inappropriate methodology, lack of detail about tools used or treatment provided. The aim of our study was to explore the effects of breathing re-education on deep neck muscles, endurance, strength and respiratory function in people with chronic neck pain.

## Method

This was a parallel group randomised controlled trial with 1:1 allocation ratio to two groups. The trial was prospectively registered in the Iranian Registry of Clinical Trials (IRCT 20200226046623N1). The trial was conducted according to the consolidated standards of reporting trial CONSORT guidelines (Schulz, Altman & Moher 2010).

Thirty patients, 14 men and 16 women (aged 25–50 years), who had non-specific neck pain for more than 3 months, participated in our study. Data were collected from the patients attending the physiotherapy department district headquarter hospital Faisalabad, Pakistan during the period August 2020 to December 2020. Patients with upper cervical symptoms (dizziness, lightheadedness) and post-traumatic neck pain were excluded due to possible influences on the outcome measures during assessment and intervention procedures (Humphreys & Peterson 2013; Ris et al. 2017). Patients with a known history of smoking, allergic asthma, depression and treatment with antidepressants were also excluded, as all of these conditions influence pulmonary function directly or indirectly (Paine et al. 2019; Park et al. 2018; Rawashdeh & Alnawaiseh 2018; Vozoris 2014a, 2014b).

A sample size of 30 was estimated using the following formula:


$$n=\frac{\left\{\left(\delta_{1}^{2}+\delta_{2}^{2}\right)\times {\left(Z_{1-\alpha /2}+Z_{1-\beta }\right)}^{2}\right\}}{{\left|\mu_{2}-\mu_{1}\right|}^{2}}$$

Here, *n* = 34 in each group, Z_1-α/2_ = standardised level of significance = 95% = 1.96, Z_1-β_ = Power of test = 80% = 1.28, µ_1_ = Mean in control group = 4.60, µ_2_ = Mean in physiotherapy group = 5.40, δ_1_^2^ = standard deviation in control group = 0.84, δ_2_^2^ = standard deviation in physiotherapy group = 0.59. The primary outcome used to estimate sample size was vital capacity (VC) (Duymaz 2019). A difference of 0.5 in the standard deviation was regarded as a meaningful change (Hislop et al. 2014).

Blocked randomisation was done using concealed allocation and patients were randomly divided into two groups, Group A and Group B in 1:1. Group A with 15 patients (eight men, seven women), mean age was 38.54 years, received physiotherapy consisting of infrared radiation (IRR) over the cervical region in prone for 10 min followed by isometric exercises for flexors and extensors of the cervical spine in supine with a 10 s hold for each muscle group; 20 repetitions were performed. The physiotherapy was followed by sham breathing exercises for 15 min. For sham breathing exercises, each patient was instructed to lie in supine and place one hand on the chest and the other hand on the belly or navel region and breathe in their normal manner. The sham intervention was chosen as the best possible similar intervention available and was not expected to reproduce the effects of breathing exercises. In Group B, there were 15 patients (six men, nine women), mean age was 38.42 years, who received both physiotherapy (as for Group A) and supervised breathing exercises focusing on proper inhalation, exhalation and chest expansion for 15 min. Each patient was instructed to place one hand on the chest and the other hand on the belly or navel region, inhale slowly through the nose for 5 s – 8 s, and exhale slowly through the mouth relaxing the chest wall and the abdomen. The duration of each session was 30 min and the total treatment time for both groups was the same. Patients in both groups received the intervention five days a week for a consecutive eight weeks. The intervention was conducted by a senior physiotherapist with more than 10 years’ experience in musculoskeletal and cardiopulmonary physiotherapy. All the outcome measures were assessed by an independent assessor blinded to group allocation.

**FIGURE 1:**
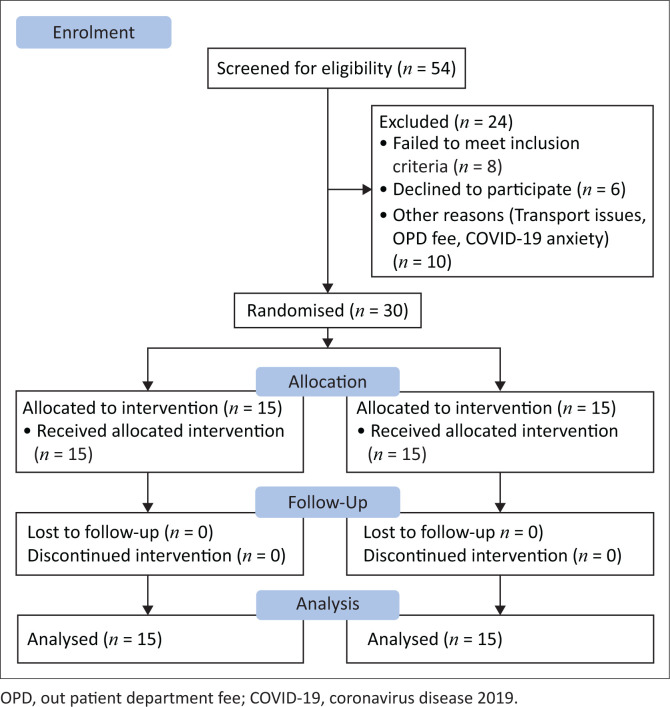
Schematic diagram of clinical trial.

Figure 1 illustrates the progression of the clinical trial.

**FIGURE 2:**
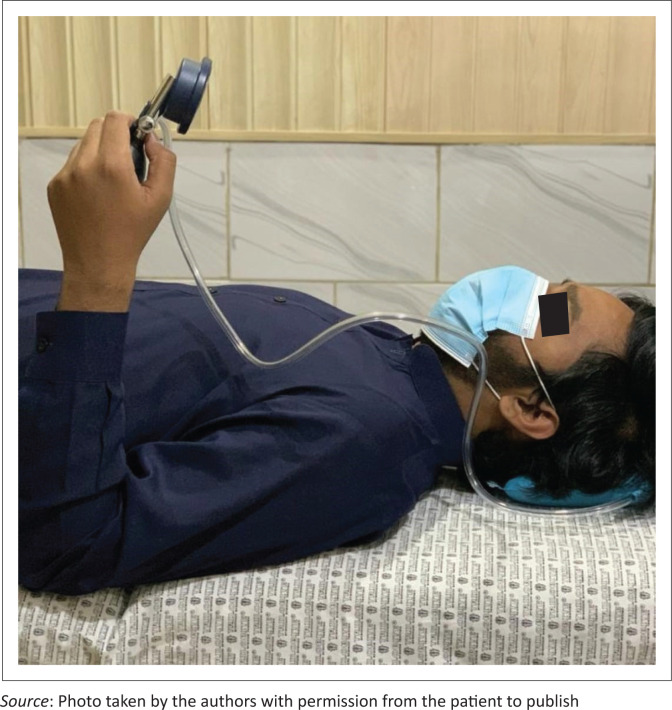
The craniocervical flexion test to measure endurance.

Cervical muscle endurance was measured with the Craniocervical Flexion Test as described by Jull et al. (2008). This is a valid and reliable test for the measurement of cervical muscle endurance (Araujo et al. 2020; James & Doe 2010). It is a low load test performed with the patient in supine. At the first stage, the patient is guided by a pressure sensor placed behind the neck. At the second stage, quantification of endurance of the neck muscles is performed. The test has five pressure levels at 22 mmHg, 24 mmHg, 26 mmHg, 28 mmHg, 30 mmHg. The patient was asked to perform a nodding action at the first level and instructed to hold it for 10 s. Reduced endurance at each level was measured by a decrease in pressure on the pressure sensor (Figure 2).

**FIGURE 3:**
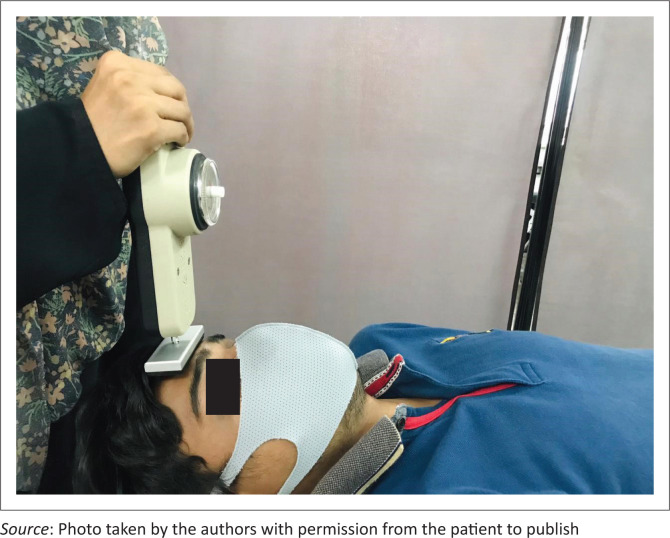
Measurement of the neck flexor strength with the handheld dynamometer.

**FIGURE 4:**
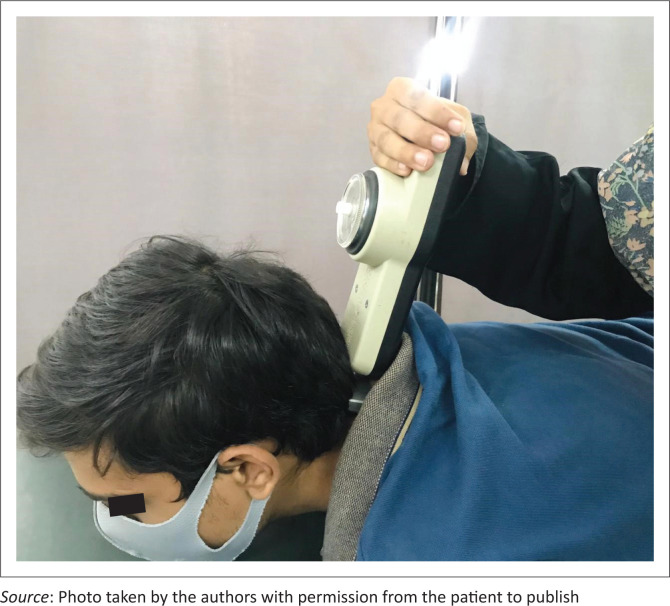
Measurement of the neck extensor strength with the handheld dynamometer.

Cervical muscle strength was measured with a handheld dynamometer (Baseline Lite 200 lb) which has good interrater and intrarater reliability (Vannebo et al. 2018). To test flexor strength, patients were placed in supine and were asked to lift their head slightly while keeping their chin tucked in (Break test). The flat head of the dynamometer was placed on the forehead to measure flexor strength (Figure 3). For extension strength, the patients were in prone, and the dynamometer was placed at the occiput and the patients were asked to lift their head slightly keeping the chin tucked in (Figure 4).

Pulmonary function was measured with spirometry with the Spirolab4 (USA). Two pulmonary tests were performed, namely, (1) VC manoeuver, and (2) Forced Expiratory Technique (FET). This manoeuver included measurement of the Forced Expiratory Volume in one second (FEV1), Forced Vital Capacity (FVC) and FEV1/FVC percentage (Levy et al. 2009; Miller et al. 2005).

### Statistical analyses

The Shapiro-Wilk test was used to test the normality of the data, and data were normally distributed. Descriptive statistics are displayed as means and standard deviations. The two-way repeated-measures analysis of variance (ANOVA) was used to analyse and compare differences between groups (Group A vs. Group B). The statistically significance level was accepted at *p* < 0.05. The data were analysed using Stat software SPSS version 21.0 (SPSS Inc., Chicago, USA).

### Ethical considerations

Our study was approved by the Ethical Review Committee of the University of Lahore, Pakistan, reference number: IRB/UOL/FAHS/697/2020. Trial registration: IRCT 20200226046623N1 https://www.irct.ir/trial/46240. After ethical approval from the University of Lahore, permission was also obtained from the Independent University Hospital Faisalabad for data collection. Written informed consent in Urdu and English was taken from each participant prior to our study.

## Results

The 30 participants completed the 8-week intervention in our study, and none were lost to follow-up. There were no significant differences between groups in the mean age and Body Mass Index (BMI), endurance, strength of neck muscles and pulmonary function. Tabular representation of baseline characteristics is given in Table 1.

**TABLE 1:** Baseline characteristics.

Variables	Group A Mean ± SD	Group B Mean ± SD
Age (Years)	38.54 ± 6.72	38.42 ± 5.12
Gender (Male/Female)	8/7	6/9
Height (cms)	158.1 ± 6.33	156.71 ± 8.26
Weight (kg)	64.85 ± 8.15	62.57 ± 8.14
BMI kg/m^2^	25.84 ± 1.51	25.37 ± 8.26

SD, standard deviation.

There was no noticeable difference between groups in the pre-intervention values of endurance, strength of cervical flexors, extensors FEV1, FVC and FEV1/FVC percentage. A two-way repeated measures ANOVA was performed to compare the effect of treatment given to Group A and Group B on endurance, strength of neck flexors and extensors, and pulmonary function.

The between-group analysis showed a statistically significant increase in the strength of the neck flexors for Group B (breathing re-education group); (F [2, 24] = 17.74, *p* = 0.0012). The endurance and strength of neck extensors had no between-group differences (F [2, 24] = 0.396, *p* = 0.3102), (F [2, 22] = 0.164, *p* = 0.3951) (Table 2).

**TABLE 2:** Intergroup comparison of variables before and after the intervention at beseline, 4th and 8th week.

Variable	Baseline		4th week		8th week		*f*	*p*
	Group A	Group B	Group A	Group B	Group A	Group B		
Endurance (mmHg)	19.14 ± 1.21	18.85 ± 1.06	21.14 ± 1.21	20.57 ± 0.97	23.57 ± 0.78	22.85 ± 1.06	0.396	0.3102
Strength Neck flexors (kg)	12.02 ± 0.81	11.42 ± 0.53	14.85 ± 1.21	13.28 ± 0.48	17.81 ± 1.06	14.85 ± 0.69	17.74	0.0012
Strength Neck extensor (kg)	11.71 ± 0.48	11.57 ± 0.97	13.71 ± 0.48	13.42 ± 0.78	15.14 ± 0.37	14.83 ± 0.75	0.164	0.3951
FEV1 L/S	2.164 ± 0.124	2.185 ± 0.06	3.37 ± 0.099	3.46 ± 0.073	3.84 ± 0.127	3.84 ± 0.127	2.475	0.830
FVC	3.25 ± 0.18	3.25 ± 0.22	3.74 ± 0.25	3.45 ± 0.22	4.12 ± 0.19	3.52 ± 0.205	52.06	0.020
FEV1/FVC%	64.17 ± 2.56	66.10 ± 3.46	71.28 ± 5.55	68.10 ± 3.16	79.28 ± 3.98	69.85 ± 2.85	35.85	0.602

Kg, kilogram; L/S, liter/second; FEV1, Forced Expiratory Volume in one second; FVC, Forced Vital Capacity.

F, Test statistics (analysis of variance with repeated measurements); *p* < 0.05.

For pulmonary function, there was a statistically significant improvement in FVC (F [2, 24] = 52.06, *p* = 0.020) for Group B (breathing reeducation group). Forced Expiratory Volume in one second and FEV1/FVC percentage had no between-group differences (F [(2, 21] = 2.475, *p* = 0.830), (F [2, 22] = 35.85, *p* = 0.602) (Table 2).

The details of the pair wise comparison of the variables at different time points are given in Table 3.

**TABLE 3:** Pair wise comparison between two groups at different time points.

Variable	Time (weeks)	Group A			Group B		
		Mean difference	95% CI for mean difference	*p*	Mean difference	95% CI for mean difference	*p*
Endurance (mmHg)	0–4	−1.85	−2.25–1.46	0.0001	−1.71	−2.65–0.77	0.0030
	4–8	−2.35	−3.19–1.52	0.0002	−2.28	−3.22–1.34	0.0010
	0–8	−4.21	3.48–4.94	0.0001	−4.00	−4.00–4.00	0.0000
Strength neck flexor (kg)	0–4	−2.34	−2.95–1.73	0.0002	−1.85	−2.32–1.38	0.0000
	4–8	−2.26	−2.75–1.77	0.0003	−1.57	−2.23–0.90	0.0010
	0–8	−4.60	−5.16–4.05	0.0000	−3.42	−4.09–2.76	0.0000
Strength neck extensor (kg)	0–4	−1.91	−2.13–1.70	0.0000	−1.83	−2.42–1.24	0.0010
	4–8	−1.46	−1.88–1.04	0.0000	−1.50	−2.29–0.71	0.0030
	0–8	−3.38	−3.91–2.85	0.0000	−3.33	−4.51–2.15	0.0010
FEV1 (L/s)	0–4	−1.24	−1.34–1.14	0.0000	−1.27	−1.42–1.12	0.0000
	4–8	−0.37	−0.49–0.28	0.0000	−0.29	−0.38–0.19	0.0000
	0–8	−1.62	−1.75–1.49	0.0000	−1.57	−1.77–1.37	0.0000
FVC	0–4	−0.34	−0.43–0.25	0.0001	−0.49	−0.70–0.27	0.0000
	4–8	−0.22	−0.29–0.16	o.0001	−0.38	−0.53–0.23	0.0000
	0–8	−0.55	−0.65–0.48	0.0000	−0.87	−1.06–0.68	0.0000
FEV1/FVC (%)	0–4	−4.28	−6.31–2.25	0.0001	−2.00	−3.01–0.98	0.0020
	4–8	−4.92	−6.39–3.46	0.0001	−1.85	−2.17–1.00	0.0010
	0–8	−9.21	−7.46–10.96	0.0001	−3.85	−4.97–2.73	0.0000

FVC, Forced Vital Capacity; FEV1, Forced Expiratory Volume in one second; CI, confidence interval.

## Discussion

Our study explored the effects of breathing re-education on the endurance and strength of the deep neck flexors and pulmonary function in patients with chronic neck pain. Our results showed that only cervical flexor muscle strength and FVC were significantly improved in Group B (breathing re-education group) as compared to Group A. The overall improved strength of cervical muscles in chronic neck pain patients may lead to less disability and improved quality of life. The improvement in VC will lead to more oxygen available to the neuromuscular system and may lead to less stress and less pain in patients with chronic neck pain.

Our results are similar to a study to observe the immediate effects of breathing re-education on pain and muscle activation of patients with neck pain (Yeampattanaporn et al. 2014). In their study, 36 patients were divided into three treatment groups and each group received breathing re-education in a different pattern. The duration of breathing re-education was 30 min and according to the results, there was a reduction in pain and superficial cervical muscle over-activity. However, all the measurements were taken in the same session immediately after the intervention. Moreover, it was unclear that among all the breathing patterns which breathing pattern was more beneficial in improving outcome measures. In our study, we observed the effects of eight weeks breathing re-education, a duration long enough to observe any significant change in the strength and endurance of cervical muscles. In another study evaluating a mobile app based self-management neck programme, the incorporation of deep slow breathing with stretching resulted in a significant improvement in pain intensity, muscle activation and pain threshold (Thongtipmak et al. 2020). It was a self management programme and patients were responsible for the execution of breathing and stretching based on the mobile app. However in this study, there is no data describing how religiously patients executed the plan, and whether the method of stretching and breathing re-education was accurate or not. In our study, supervised breathing re-education along with a superficial heating technique and isometric exercises for consecutive 8 weeks resulted in improved strength of deep neck flexors and improved FVC. Wirth et al. (2014) investigated the relationship of chest mobility and respiratory function with neck disability, in 19 healthy people and 19 people with chronic neck pain. The mobility of the thoracic spine during flexion and chest expansion showed a significant moderate correlation with maximum voluntary ventilation (MVV) (*r* = 0.45 and 0.42) and neck muscle endurance (*r*_S_ = 0.36) (Wirth et al. 2014). Based on their findings they suggested that people with chronic neck pain should improve endurance of their neck muscles and chest mobility. Accordingly, in our study we observed the difference in endurance of neck flexors in the control group from 18.85 ± 1.06 at baseline to 22.85 ± 1.06 after eight weeks and the experimental group from 19.14 ± 1.21 at baseline to 23.57 ± 0.78 after eight weeks of intervention. However, there was no significant between group difference (*p* = 0.3102).

In another study, breathing re-education combined with neck stabilising exercises resulted in improvement of respiratory function in patients with stroke. In the said study, 45 patients with stroke who were divided into three groups underwent a six-week breathing re-education programme. Outcome measures were deep neck flexor thickness measured through electromyography and respiratory function measured through spirometry (Lee & Hwang-Bo 2015). Based on their findings it was evident that the treatment group with a combination of neck stabilising exercises and breathing re-education, showed more improvement in pulmonary function as compared to the breathing re-education only group. One important finding of the study was that the deep neck flexor thickness was improved only in the combination group. Our study showed similar results. Although the patient population was different, the breathing re-education combined with the cervical isometrics showed improvement in pulmonary function. A limitation of the above study was that only deep neck flexor thickness was assessed. In our study we assessed the strength and the endurance of the neck muscles. Our results explain the dual role of cervical muscles as prime movers of the neck and as accessory muscles of the respiration.

Kang et al. (2016) investigated the activities of sternocleidomastoid and anterior scalenus muscle by implementing breathing exercises in 30 individuals with forward head posture. They concluded that these muscles as accessory muscles of respiration can add to respiratory inefficiency if they are weak, overactive, or tight. The muscles of the participants in the intervention group were more efficient after a feedback breathing programme as measured through electromyography. The results of this study are similar to our findings where a breathing re-education regime resulted in improved cervical flexor strength (Kang et al. 2016).

In another study on 40 healthy men, breathing exercises combined with dynamic upper extremity exercises resulted in improved FVC and FEV1. The 40 participants were divided into two groups, both groups received breathing exercises and the experimental group performed breathing exercises combined with dynamic upper extremity exercises. It was a four-week intervention programme and FVC increased significantly in both groups but between group analysis showed there was no difference in FEV1 before and after the intervention (Han & Kim 2018). In our study the breathing re-education programme showed similar results. Thus, it can be speculated that people with chronic neck pain can benefit from breathing re-education and it may help in improving their respiratory efficiency and decreasing the risks of comorbidities related to it.

The overall findings of our study showed that breathing re-education resulted in improved cervical flexor muscle strength and improved FVC. This regime may be used as an adjunct treatment with superficial heat and cervical muscle strengthening in people with chronic neck pain.

### Limitations

One of the limitations of our study was that we only measured FEV1 and FVC. Measurement of other parameters of pulmonary function such as total lung capacity and residual volume may give more insight into the effect of breathing re-education on pulmonary function in people with chronic neck pain. Another limitation is the treatment duration, that is, five days a week for eight weeks. The treatment duration was difficult for the patients to comply with, and this may present a barrier to implementation. Our study was conducted in a single setting only; multicentre large-scale trials are needed to consider breathing re-education as a regular part of the multimodal treatment programme in the treatment of chronic neck pain.

## Conclusion

In conclusion, our findings suggest that breathing re-education combined with other physiotherapy management improves strength of neck flexors and FVC in people with chronic neck pain. Good respiratory technique and improved strength of neck flexors may lead to less fatigue, less pain experienced and possibly improved quality of life in these patients. Thus, breathing re-education may be an important regime to include in the treatment of people with chronic neck pain.
