# Corrigendum: Effects of breathing re-education on endurance, strength of deep neck flexors and pulmonary function in patients with chronic neck pain: A randomised controlled trial

**DOI:** 10.4102/sajp.v79i1.1793

**Published:** 2023-05-09

**Authors:** Sahreen Anwar, Syed A. Arsalan, Hamayun Zafar, Ashfaq Ahmed, Syed A. Gillani, Asif Hanif

**Affiliations:** 1 University Institute of Physical Therapy, University of Lahore, Lahore, Pakistan; 2 Faculty of Rehabilitation Sciences, King Saud University, Riyadh, Saudi Arabia; 3 Faculty of Allied Health Sciences, University of Lahore, Lahore, Pakistan; 4 University institute of Public Health, University of Lahore, Lahore, Pakistan

In the published article, Anwar, S., Arsalan, S.A., Zafar, H., Ahmed, A., Gillani, S.A. & Hanif, A., 2022, ‘Effects of breathing re-education on endurance, strength of deep neck flexors and pulmonary function in patients with chronic neck pain: A randomised controlled trial’, *South African Journal of Physiotherapy* 78(1), a1611. https://doi.org/10.4102/sajp.v78i1.1611, there was an error regarding the affiliations for the authors. Instead of:

‘**Authors:**

Sahreen Anwar^1^https://orcid.org/0000-0002-6603-3185

Syed A. Arsalan^1^https://orcid.org/0000-0003-3492-7050

Hamayun Zafar^1,2^https://orcid.org/0000-0001-9318-3691

Ashfaq Ahmed^1^https://orcid.org/0000-0002-1965-6224

Syed A. Gillani^3^https://orcid.org/0000-0002-2996-0764

Asif Hanif^4^https://orcid.org/0000-0002-2670-6402

**Affiliations:**

^1^Department of Physical Therapy, Faculty of Rehabilitation Sciences, University of Lahore, Lahore, Pakistan

^2^Department of Physical Therapy, Faculty of Rehabilitation Sciences, King Saud University, Riyadh, Saudi Arabia

^3^Department of Biostatistics, Faculty of Rehabilitation Sciences, University of Lahore, Lahore, Pakistan’

It should be:

**‘Authors:**

Sahreen Anwar^1^https://orcid.org/0000-0002-6603-3185

Syed A. Arsalan^1^https://orcid.org/0000-0003-3492-7050

Hamayun Zafar^1,2^https://orcid.org/0000-0001-9318-3691

Ashfaq Ahmed^1^https://orcid.org/0000-0002-1965-6224

Syed A. Gillani^3^https://orcid.org/0000-0002-2996-0764

Asif Hanif^4^https://orcid.org/0000-0002-2670-6402

**Affiliations:**

^1^University Institute of Physical Therapy, University of Lahore, Lahore, Pakistan

^2^Faculty of Rehabilitation Sciences, King Saud University, Riyadh, Saudi Arabia

^3^Faculty of Allied Health Sciences, University of Lahore, Lahore, Pakistan

^4^University Institute of Public Health, University of Lahore, Lahore, Pakistan’.

In addition, there was an error on page 2. The following paragraph is updated as it was incorrectly formulated:

**The original incorrect wording:**

Here, *n* = 34 in each group, Z_1-α/2_ = standardised level of significance = 95% = 1.96, Z_1-β_ = Power of test = 80% = 1.28, µ_1_ = Mean in control group = 4.60, µ_2_ = Mean in physiotherapy group = 5.40, δ_1_^2^ = standard deviation in control group = 0.84, δ_2_^2^ = standard deviation in physiotherapy group = 0.59. The primary outcome used to estimate sample size was vital capacity (VC) (Duymaz 2019). A difference of 0.5 in the standard deviation was regarded as a meaningful change (Hislop et al. 2014).

**The revised and updated wording:**

Here, *n* = 15 in each group, Z_1-α/2_ = standardised level of significance = 95% = 1.96, Z_1-β_ = power of test = 80% = 1.28, µ_1_ = mean in control group = 4.60, µ_2_ = mean in physiotherapy group = 5.40, δ_1_^2^ = standard deviation in control group = 0.84, δ_2_^2^ = standard deviation in physiotherapy group = 0.59. The primary outcome used to estimate sample size was vital capacity (VC) (Duymaz 2019). A difference of 0.5 in the standard deviation was regarded as a meaningful change (Hislop et al. 2014).

The authors apologise for these errors. The corrections do not change the study’s findings of significance or overall interpretation of the study’s results or the scientific conclusions of the article in any way.

